# Neurotransmitter Systems Affected by PBDE Exposure: Insights from In Vivo and In Vitro Neurotoxicity Studies

**DOI:** 10.3390/toxics13040316

**Published:** 2025-04-18

**Authors:** Wendy Argelia García-Suastegui, Cynthia Navarro-Mabarak, Daniela Silva-Adaya, Heidy Galilea Dolores-Raymundo, Mhar Yovavyn Alvarez-Gonzalez, Martha León-Olea, Lucio Antonio Ramos-Chávez

**Affiliations:** 1 Departamento de Biología y Toxicología de la Reproducción, Instituto de Ciencias, Benemérita Universidad Autónoma de Puebla, Puebla C.P. 72570, Mexico; wendy.garcias@correo.buap.mx (W.A.G.-S.); hgali88@hotmail.com (H.G.D.-R.); 2 División de Neurociencias, Instituto de Fisiología Celular, Universidad Nacional Autónoma de Mexico, Mexico City C.P. 04510, Mexico; cnvmabarak@yahoo.com.mx; 3 Laboratorio Experimental de Enfermedades Neurodegenerativas, Instituto Nacional de Neurología y Neurocirugía, Ciudad de Mexico 14269, Mexico; adayadani@gmail.com; 4 Centro de Investigación Sobre el Envejecimiento, Centro de Investigación y de Estudios Avanzados (CIE-CINVESTAV), Ciudad de Mexico C.P. 14330, Mexico; 5 Departamento de Neuromorfología Funcional, Dirección de Investigaciones en Neurociencias, Instituto Nacional de Psiquiatría Ramón de la Fuente Muñiz, Ciudad de Mexico C.P. 14370, Mexico; mhar_ag@inprf.gob.mx

**Keywords:** polybrominated diphenyl ethers, neurotoxicity, neurotransmitters

## Abstract

Polybrominated diphenyl ethers (PBDEs) are synthetic halogen compounds, industrially used as flame retardants in many flammable products. PBDEs are environmentally persistent and bioaccumulative substances that were used from the 1970s and discontinued in the 1990s. PBDEs are present in air, soil, water, and food, where they remain stable for a long time. Chronic exposure to PBDEs is associated with adverse human health effects, including cancer, immunotoxicity, hepatotoxicity, reproductive and metabolic disorders, motor and hormonal impairments, and neurotoxicity, especially in children. It has been demonstrated that PBDE exposure can cause mitochondrial and DNA damage, apoptosis, oxidative stress, epigenetic modifications, and changes in calcium and neurotransmitter levels. Here, we conduct a comprehensive review of the molecular mechanisms of the neurotoxicity of PBDEs using different approaches. We discuss the main neurotransmitter pathways affected by exposure to PBDEs in vitro and in vivo in different mammalian models. Excitatory and inhibitory signaling pathways are the putative target where PBDEs carry out their neurotoxicity. Based on this evidence, environmental PBDEs are considered a risk to human public health and a hazard to biota, underscoring the need for environmental monitoring to mitigate exposure to PBDEs.

## 1. Overview of Polybrominated Diphenyl Ethers

Polybrominated diphenyl ethers (PBDEs) are chemicals used industrially to avoid burning flammable products by delivering bromine (Br) radicals with heat. In fact, the use of PBDEs has helped to significantly decrease accidental fires (~64% in USA) [1,2]. PBDEs are a group of additive compounds noncovalently bound to different commercial products with differences in physical, chemical, and biological activity due to the number of Br substitutions (Table 1). These compounds are classified according to the number of Br atoms added to the hydrocarbon backbone molecule. A radical group of up to 10 bromines has been described with chemical formula C12H(9–10)Br(1–10)O. There are now approximately 209 commercial PBDE congeners (PBDEs 1-209), grouped into 10 subgroups, mono- to decabrominated diphenyl ethers (BDEs) [3,4,5]. Nowadays, the decaBDE group is the most commercially used; however, in the presence of light, it is degraded (photolytic degradation) to tetra-, penta-, or hexaBDEs, which have more cumulative capabilities in animal fat tissues (food biomagnification) due to their low molecular weight [3,6,7]. PBDE biodegradation is possible, leading to a PBDE mixture with greater environmental bioavailability [8]. Furthermore, Br atoms in BDEs influence their environmental availability; PBDEs with more Br atoms have minor distribution, volatilization, and hydrophobic properties [9].

In the environment, air and water are the main transport routes of PBDE congeners, which can travel long distances from their source (>1000 km) [10]. Since 2009, PBDEs have been listed as Persistent Organic Pollutants in the Stockholm Convention. Chemically, PBDEs are semi-volatile compounds highly resistant to degradation (half-lives of 2–10 years). Indeed, PBDEs have lipophilic properties and are insoluble in water (<1 μg/L) due to Br radicals [10]. PBDEs are stored in fat tissue cells with a half-life of ~4 years in humans [11,12,13,14]. The most common PBDE congeners found in humans, food, household dust, and wild animals are tetraBDE-47, followed by pentaBDE-99 and hexaBDE-153 [9]. PBDE congeners are resistant to physical, chemical, and biological degradation; however, PBDEs undergo hepatic metabolism in mammalians through xenobiotic metabolizing enzymes, cytochrome P450 monooxygenases (CYP) 1A2, 3A4, and reductive enzymes (deiodinase type 1, and glutathione S-transferase M1) [4,7,15].

Currently, millions of products, mainly electronics, contain PBDE mixtures (~30% weight), mainly deca-, octa- and pentaBDE mixtures. Compounds with tetra- or minor Br radicals are not industrially used [3,5,9]. The commercial products that contain the most PBDEs are textiles, polyurethane foams, furniture upholstery, automobile parts, carpets, and electronics like televisions and computers, among others, where they undergo volatilization for a long time [2,4,5,9,16]. North America, Israel, and Asia produce ~95% (~80 kilotonnes/year in 2000) of PBDE mixtures (penta-, octa-, and decaBDE), and the rest of the world ~5 kilotonnes/year. Since 2000, the use of PBDEs in production has declined [17]. In 2008, the U.S. EPA established that a safe daily level of exposure to PBDE congeners ranges from 0.1 to 7 µg/kg body weight/day for the four the most common PBDE congeners (BDE-99, -47, -153, and -209). The European Union has a higher limit of 1 g/kg for PBDE exposure. In fish, the standard limit of total PBDE levels is 0.0085 µg/kg of weight (Directive 2013/39/EU). In fact, fish is the main source of human exposure, but outdoor dust is another important source of PBDEs [18,19].

In humans, the routes of PBDE entry are oral, respiratory, and dermal [20,21]. Epidemiologically, oral ingestion is the most relevant human route of exposure to PBDEs [22]. It is unknown how many people are chronically exposed to PBDEs. However, it has been reported that levels of human exposure to BDE-209 are greater than 3000 ng/g lipid for Chinese adult inhabitants in areas near electronic waste dumps [23,24]. PBDEs have been detected in birds, marine animals, and human tissue such as adipose tissue, blood, and maternal milk, reflecting their widespread occurrence in different environmental compartments. Indeed, PBDEs are bioaccumulated in the food chain and persist in the environment at higher levels than other banned persistent pollutants [3,4].

Neuroendocrine disruption is the classical target associated with chronic exposure to PBDEs. Like polychlorinated compounds, polybrominated compounds affect the balance in thyroid hormones, testosterone, and estrogen levels. Structurally, PBDEs resemble thyroid hormones (xenohormonal proprieties). PBDEs alter the synthesis, secretion, transport, binding, activity, and elimination of hormones involved in development, reproduction, behavior, and fertility. Hormonal disorders are observed in offspring from mothers with PBDE gestation exposure. Thyroid hormones like T4, FT4, T3, and TSH are decreased by exposure to PBDEs [4,25]. Psychomotor damage, intellectual disability, and reproductive damage are observed in mammalian models of PBDE intoxication. In U.S. adult males, PBDEs are associated with increased thyroglobulin antibodies and T4, and testosterone levels [19]. Besides hormonal alterations, PBDEs’ effects on human health are diverse. In adults, exposure to PBDEs is associated with cancer, hepatotoxicity, immunotoxicity, reproductive damage, motor damage, and neurotoxicity. The effects PBDEs, like many other toxic compounds, depend on sex, time, and level of exposure. Particularly, children have the highest grade of exposure to PBDEs. Fetal exposure to PBDEs is associated with fetal growth restriction [26], reproductive damage [27], and lower IQ scores [28]. Moreover, the congener type of PBDEs is related to their effect; tetra- to hexa-PBDEs are more carcinogenic, more hormonally disruptive, and have more neurotoxic potential (Table 1; [1,29]). Table 2 summarizes neurological findings associated with human exposure to PBDEs during the prenatal, childhood, and adult stages. Most studies focus on neurodevelopmental alterations or early disruptions in brain function due to PBDE exposure and long term and irreversible effects on health and the quality of life.

Recently, it was pointed that the neurotoxicity of PBDEs is associated with disruptions in the neurotransmitter system, increased oxidative molecules, alterations in neurotransmitter transport and receptor binding, and impaired synaptic signaling [29]. Here, we conduct a comprehensive review of the molecular mechanisms of the neurotoxicity of PBDEs using different approaches.

## 2. Methodology

In this review, we searched the PubMed and Google Scholar databases. Keywords including PBDE congeners, toxicity, brain, metabolism, memory, neurotoxicity, neurons, neurotransmitters, glutamate, gamma-aminobutyric acid (GABA), dopamine, acetylcholine, and nitric oxide were used in the search. For each search, 2 keywords were used. We reviewed all studies published to date. A total of 2463 toxicity studies were identified in online databases. Then, we highlighted the relevance of toxicity metabolism and neurotoxicity and impairment of PBDE neurotransmitters, so 590 works were selected, of which 87 were selected for the analysis of different neurotransmitters, of which 27 studies included changes in different neurotransmitters.

## 3. Toxicity and Metabolism of PBDEs

The biotransformation of PBDEs is not completely described in mammals [30,31]. However, it has been reported that liver metabolism is involved in the toxicity of PBDEs. Studies in adult rodents exposed to tetraBDE-47, pentaBDE-99, pentaBDE-100, hexaBDE-153, and hexaBDE-154 have shown that PBDEs were absorbed in more than 80% [15,30]. Following absorption, the PBDEs are differentially distributed in tissues but are accumulated mainly in adipose tissue (16–22%), skin (6.4–7.1%), muscle (5.2–7.6%), and liver (1.4–2.1%). In rodents, the half-life of these compounds is 72 h after oral administration, with ~40% excreted in feces and <1% in urine [15].

The toxicity of PBDEs is mainly due to their chemical structure and the products of their enzymatic metabolism. The clearance of PBDEs depends on the number of bromine atoms; the greater the number of Br radicals, the lower their elimination is [3]. In vitro and in vivo studies have shown that some PBDE congeners are substrates of cytochrome P450 monooxygenases (CYP) 1A2, CYP3A4, and glutathione S-transferase M1, resulting in mono-hydroxylated, di-hydroxylated, and thiol metabolites excreted mostly in feces (~90%). The products of this enzymatic metabolism are more toxic to cells. However, bromine breakdown has not been completely described [15,31]. Other PBDE derivatives have shown to be neurotoxic, such as Quinone-PBDEs (QPBDEs), as observed in rodents exposed to PBDEs. The cytotoxicity and genotoxicity of QPBDEs in liver and neural cells has been linked to oxidative damage [32,33,34]. For example, polybrominated metabolites, such as brominated phenol PBDEs (2,4-DBP and 4-BP) and hydroxylated PBDE metabolites (6-OH-BDE-47, 5-OH-BDE-47, 2-OH-BDE-28, and 4-OH-BDE-17), have a highly toxic effect on HepG2 cells, demonstrated by an increase in reactive oxygen species (ROS) production, antioxidant enzyme dysfunction, DNA damage, and apoptosis [35]. Similarly, BDE-47 derivatives such as 6-OH-BDE-47 and 6-MeO-BDE-47 decreased cell viability and increased apoptosis, genotoxicity, and biomarkers of oxidative stress [36]. Additionally, hydroxylated PBDEs (OH-PBDEs), as well as2-OH-BDE-47 and 2-OH-BDE-85, are toxic at µM concentrations, in a dose-dependent manner [37].

Further different studies reported PBDEs’ effects in other tissues like the brain, where the biotransformation of PBDEs is unknown. The metabolism of PBDEs depends on congener type, species, life stage, and route and time of exposure. In fact, exposure to PBDEs is chronic and ubiquitous and occurs in all life stages, including gestation, during which sensitivity to PBDEs is greater, with long-term and irreversible implications for health (Table 2) [38].

**Table 2:** Neuropathological effects in children and adults exposed to PBDEs more frequently detected in the environment (BDE-17, -28, -47, -99, -100, -153, -154, and -183).

Measured BPDEs	Topic	Population/Exposure	Neurological Finding	Reference
Children				
BDE-47, -99, -100, and -153	Autism spectrum disorder	154 (36 months of age), Philadelphia, PA; Baltimore, MD San Francisco Bay Area, CA; and Sacramento, CA, USA—Pregnancy at 2rd trimester or the 3rd trimester.	BDE-47 (5.9–19.2 ng/g in umbilical cord) was associated with greater deficits in social reciprocity (β = 6.39, 95% CI: 1.12, 11.65).	[39]
BDE-28, -47, -99, -100, -153, -154, and -183	Executive functions in adolescents	115, (12–18-year-old, 53 male and 62 female), Green Bay, Wisconsin area, USA—Two weeks after the neuropsychological assessment.	BDE-47 and BDE-153 (median serum total PBDE 29.14 ng/g of serum lipid) were associated with poorer cognitive flexibility	[40]
BDE-47, -99, -100, and -153	Cognitive and psychomotor development	355 (6–8 years of age), Quebec, Canada—Early pregnancy (12 weeks of gestation) and at delivery.	Decrease in spatial perception and reasoning was associated with higher BDE-100 (0.019 +/− 0.052 in blood) concentration at delivery.	[41]
BDE-47	Placental epigenetic and neurodevelopment	260 pregnant women with 10 to 13.14 weeks, from 12 clinic sites within the USA—First trimester of pregnancy.	BDE-47 (3.60, 16.67 ng/g lipid) chance-methylated CpG sites in pathways related to brain size and brain morphology and with birth weight (r = −0.16, *p* value = 0.01) and head circumference (r = −0.16, *p* value = 0.01).	[42]
BDE-17, -28, -47, -66, -85, -99, -100, -153, -154, -183, and -209	Intrinsic functional network organization	34, (5-year-old children), New York City—First half of pregnancy (12.2 weeks gestation, SD = 2.8 weeks).	PBDE serum concentrations correlated with higher global efficiency of brain areas involved in visual attention (VA); VA was associated with more executive functioning problems (β’s = 0.01, FDR-corrected p’s < 0.05).	[43]
BDE-28, -47, -99, -100, -153, -154, and -183	Preschool maturity	91, (6-year-olds), Eastern Slovakia—6-years old.	Negative associations of BDE-153 (*p* = 0.002, b = −29.8) and WPPSI-III composite score. Adverse effects on preschool maturity and neuropsychological development.	[44]
BDE-15, -17, -25, -28, -33, -47, -99, -100, and -153	Frustration in infancy	333, (6 to 7 months), Canada—First trimester of pregnancy.	Predisposition to frustration and lack of habituation and BDE-47 (7.32–727.3 ng/g lipid) was associated with negative vocalizations (adjusted Relative Risk [aRR] = 1.04, 95% CI: 1.00, 1.09).	[45]
BDE-47, -99, -100, -153, -154, and HBCD	Development at adolescence	101, (55 boys and 46 girl), Western European—Second and/or third trimester of pregnancy.	BDE-154 was negatively associated with verbal memory recall (−0.303 *p* = 0.07) and delayed recognition (−0.348 *p* = 0.041) impairment. BDE-153 was negatively associated with auditory attention (−0.379 *p* = 0.03).	[46]
BDE-17, -28, -47, -66, -85, -99, -100, -153, -183, and -209	Visual spatial abilities	199 (8 years), Cincinnati area (OH, USA)—16 ± 3 weeks gestation, 1 year, 2 years, 3 years, 5 years, and 8 years.	Impairments in visual spatial learning with early childhood BDE-153.	[47]
BDE-17, -28, -47, -66, -85, -99, -100, -153, -154, and -183	Intelligence Quotient and externalizing behavior problems	239, (8 years old), Cincinnati, OH, USA—16 ± 3 weeks of gestation	Associated with the score for externalizing behavior problems (β: 3.5, 95% CI: −0.1, 7.2) at age 8 years.	[48]
BDE-28, -47, -99, -100, -153, -154, -183 and -209	Neurodevelopment	246 (6-year-old child), Brittany region, France—On partum.	Verbal comprehension scores were lower in children from homes with higher concentrations of BDE-99 or -209.	[49]
BDE-47, -85, -99, -100, -153, -154, and -183	Early childhood attention problems	210, (3 and 7 years), downtown New York City—At the time of delivery.	Attention problems were associated with BDE-47 (1.21, 95% CI: 1.00, 1.47) and BDE-153 (1.18, 95% CI: 1.00, 1.39) at age 4 years.	[50]
BDE-47, -99, -100, and -153	Attention and executive function	301, (9 to 12 years old), Salinas Valley CA, USA—~26 weeks gestation (M = 26.7, SD = 2.6 weeks gestation) or upon delivery.	Poorer response consistency on the Conners’ Continuous Performance Test II (β = 2.9; 95% CI: 0.9, 4.8) and poorer working memory on the Behavioral Rating Inventory of Executive Function (β = 2.5; 95% CI: 0.5, 4.4).	[51]
BDE-47, -99, -100, and -153	Neurodevelopmental measure (motor, language, adaptive, and social domains)	132, and 149 (12 and 24 months), Shandong province, northern China—At partum.	BDE-99 levels were associated with a 2.16-point decrease [95% confidence interval (CI): −4.52, −0.20] in language domain DQs. BDE-47 levels were associated with a 1.89-point decrease (95% CI: −3.75, −0.03) in social domain developmental quotients at 24 months of age	[52]
BDE-17, -28, -47, -66, -85, -99, -100, -153, -154, and -183	Cognitive abilities and hyperactivity behaviors	309, (1, 2, 3, 4, and 5 years of age), Cincinnati, OH, USA—16 weeks of gestation.	Prenatal BDE-47 decrease of 4.5-points in Full-Scale IQ (95% CI: −8.8, −0.1) and a 3.3-point increase (95% CI: 0.3, 6.3) in the hyperactivity score at age 5 years.	[53]
BDE-17, -28, -47, -66, -85, -99, -100, -153, -154, and -183	Children’s attention, motor functioning, and cognition	551, (5 and 7 years), Salinas Valley, CA, USA—Pregnancy (mean = 26.7 ± 2.6 weeks gestation, *n* = 219) or at delivery (*n* = 60), and from children at the 7-year visit (*n* = 272).	PBDE concentrations were associated with impaired attention as measured at 5 and 7 years of age, with poorer fine motor coordination, and with decrements in Verbal and Full-Scale IQ at 7 years.	[54]
BDE-47, -99, -100, 153, -154, -183, and -209	Mental and psychomotor development	290, (12–18 months of age), Gipuzkoa, Basque Country; and Sabadell, Catalonia—In the first 48–96 h postpartum.	BDE-209 (0.04 to 6.49 ng/g lipid) association with mental development score became slightly weaker (β = −2.10, 95% CI: −4.66, 0.46).	[55]
BDE-47, -99, -100, -153, -154, and HBCD	Motor, cognitive, and behavioral outcome	62, (5–6 years old), northern provinces of the Netherlands—35th week of pregnancy.	Brominated flame retardants correlated with worse fine manipulative abilities, worse attention, better coordination, better visual perception, and better behavior.	[56]
**Adults**				
DE-47	Post-partum depression (PPD)	367 asymptomatic pregnant women (29- to 33-year-olds), Southern California, USA—First trimester.	Exposure in the first trimester increase the PPD by 22% (OR = 1.22, 95% CI: 1.03, 1.47).	[57]
BDE-28, -47, -66, -85, -99, -100, -138, -153, and -154	Neuropsychological effects and synergy effects with PCBs	144 (67 men and 77 women of 55–74 years of age), New York, USA.	PBDEs (∑PBDEs 4.72 to 1590 ng/g) and PCBs may interact to affect verbal learning and memory.	[58]

Below is the chemical name of each PBDE congeners mentioned in the table: BDE-17: 2,2′,4-tribromodiphenyl ether; BDE-28: 2,4,4′-tribromodiphenyl ether; BDE-47: 2,2′,4,4′-tetrabromodiphenyl ether; BDE-66: 2,3′,4,4′-tetrabromodiphenyl ether; BDE-85: 2,2′,3,4,4′-pentabromodiphenyl ether; BDE-99: 2,2′4,4′,5-pentabromodiphenyl ether; BDE-100: 2,2′,4,4′,6-pentabromodiphenyl ether; BDE-138: 2,2′,3,4,4′,5′-hexabromodiphenyl ether; BDE-153: 2,2′,4,4′,5,5′-hexabromodiphenyl ether; HBCD: 1,2,5,6,9,10-hexabromocyclododecane; BDE-154: 2,2′,4,4′,5,6′-hexabromodiphenyl ether; BDE-183: 2,2′,3,4,4′,5′,6-heptabromodiphenyl ether; BDE-209: 2,2′,3,3′,4,4′,5,5′,6,6′-decabromodiphenyl ether.

## 4. Neuronal Oxidative Stress by PBDE Exposure

Neuronal integrity depends on cellular biomolecules maintained under a controlled redox status. Exposure to PBDEs disrupts redox homeostasis. PBDEs influence mostly glutathione (GSH, main brain antioxidant) bioavailability and compromise cellular structure. In vitro and in vivo studies have reported that PBDEs induce the production of reactive oxygen species (ROS) and oxidative damage, apoptosis, and mitochondrial dysfunctions [59,60,61]. However, the toxicological mechanism of PBDEs is poorly understood, but oxidative damage is mainly involved in PBDE-induced neurodysfunction [59,62,63]. In vitro and in vivo studies of PBDEs and the role of oxidative stress in their mechanism of action are described in the following sections.

### 4.1. In Vivo PBDE Exposure and Oxidative Injuries

ROS production is proposed as the primary neurotoxic mechanism caused by in vivo exposure to PBDEs [64,65]. Cerebellar cells are more vulnerable to oxidative damage by exposure to pentaBDE during adulthood (pentaBDE-99, administered by gavage in single doses of 0, 0.6, or 1.2 mg/kg/body weight) [65]. Similarly, reductions in GSH reductase activity in erythrocytes and increase urinary isoprostane levels (biomarkers of biochemical and oxidative stress) were observed after exposure to pentaBDE-99 in adult mice with nephrotoxicity and hepatotoxicity [66]. Vagula et al., 2011, reported reductions in sciatic nerve conduction and tissue pro-oxidant environment in mice acutely treated with pentaBDE-85 (i.p., 0.25 mg/kg mice for 4 days and isolated sciatic nerves of rats exposed to 5 µg/mL or 20 µg/mL of pentaBDE-85) [67]. These alterations may be related to long-term motor and cognitive impairment due to PBDEs.

Also, early PBDE effects have been investigated. In mice orally exposed to 10 mg/kg PBDE at postnatal day (PND) 10, oxidative stress and behavioral alterations were observed, but no differences in thyroid hormone levels were noted; deficiency in GSH synthesis signaled PBDE-induced neurotoxicity [59,68].

### 4.2. In Vitro PBDE Exposure and Oxidative Injuries

In vitro, it was demonstrated that at 24 h post-exposure, tetraBDE-47 decreased cell viability in SK-N-SH neuroblastoma cells in a dose-dependent manner, suggesting a putative neurotoxic mechanism [69]. In normal human hepatocytes, L02 tetraBDE-47 metabolites (3-OH-BDE47, 3-MeO-BDE47, 5-OH-BDE-47, and 5-MeO-BDE-47) decreased GSH and increased the antioxidant enzyme superoxide dismutase (SOD). 6-OH-BDE-85 decreased SOD activity and GSH level and induced cytotoxicity [62]. Similarly, in isolated cells, the effect of tetraBDE-47 on oxidative stress and apoptosis generation is more severe in neurons and astrocytes from cerebellum, hippocampus, cerebral cortex, and cerebellar granule neurons with low levels of GSH [59,70]. In addition to SOD and GSH alterations, BDE-209, -47 induces oxidative damage and apoptosis, genotoxicity, and endoplasmic reticulum stress in primary fetal hippocampal neurons and second messenger (calcium concentration) interference, and decreases global gene DNA methylation in HT-22 cell lines and neuron cultures [71,72,73].

Mitochondria, the central energy organelle and source of ~90% of ROS, is proposed as a target of oxidative damage from toxicants such as PBDEs. Antioxidant and histological damage associated with oxidative stress have been documented in Barbus graellsii fish from PBDE-contaminated rivers; depleted brain cholinesterase activity and macrophages and their aggregates were observed in several tissues [74]. However, in isolated rat liver mitochondria, pentaBDE-100 congeners deregulated calcium homoeostasis and ATP content and led to mitochondrial swelling, without increasing indicators of oxidative stress (ROS accumulation, NAD(P)H oxidation, GSH/GSSH, and -SH protein content) [75].

The brain has systems to maintain redox balance. To date, antioxidants such as GSH, thioredoxin, lipoic acid, and some vitamins have been described to prevent oxidative damage to neurons and glial cells. In fact, antioxidants like melatonin and N-acetylcysteine (GSH precursor) prevent the oxidative, proinflammatory, and cognitive injuries caused by decaBDE-209 [76,77]. In the same vein, MAPK inhibition or the Ca^2+^ inhibitor BAPTA-AM prevented BDE-47 exposure-dependent biomarker stress and apoptosis [78]. Indeed, neurotransmitter levels have been reported to influence ROS levels. Different neurotransmitter systems are involved in neuroprotection, neurodegenerative diseases, and neurotoxicity by chemicals [79]. Oxidative stress is suggested to contribute to subtle structural alterations in synapses and neurotoxicity in the developing and adult brain of animals exposed to PBDEs [29,65].

## 5. Neurotoxicity from PBDE Exposure

PBDEs and their metabolites are harmful to neurons. Brain histological studies have shown that exposure to PBDE congeners decreased the number and viability of hippocampus neurons. Neuronal death by PBDEs is related to calcium overload, oxidative stress, and overactivation of neurotransmitter systems [80]. In the next paragraphs, in vivo and in vitro neurotoxic findings on PBDEs are described.

### 5.1. In Vivo PBDE Exposure and Neurotoxicity

PBDE congeners induce changes in brain morphology and synaptic structure. Decabromodiphenyl ether (DecaBDE) exposure significantly reduced thyroid hormone and glia density from the stem cell niche in the subgranular zone in C57BL6/J mouse, where no sex effects were observed (30 mg/kg of BDE-71 for 30 days); it also impaired neuroexcitability, which is vital to brain communication and function [81]. Acute PBDE exposure in adult male Wistar rats through intraperitoneal injection of two compounds (BDE-71 and HBCD) led to an atypical increase in apoptosis 72 h after injection, where enzymatic PBDE metabolism is involved [82]. Meanwhile, neuronal migration and the dendritic development of newborn olfactory granule cells were impaired in mice exposed to decaBDE-209 from gestational day 6 to post-natal day (PND) 16 [83]. In the same line, a single oral dose of tetraBDE-47 (0, 1, 5, 10 mg/kg) at PND 10 induced long-term hippocampal apoptosis and enhanced developmental neurotoxicity, and ultrastructure analysis showed a swollen endoplasmic reticulum and degranulation [84]. Likewise, in whole-gestational exposure to decaBDE-209, autophagy was increased and hippocampal neuron viability was decreased. Spatial learning and memory were also decreased, as evaluated using the Morris water maze task [85].

### 5.2. In Vitro PBDE Exposure and Neurotoxicity

In vitro PBDE neurotoxicity has been linked to some stress biomarkers. In neurons, PBDE congeners can be accumulated in the microsomal fraction and mitochondria, where a significant amount of ROS is produced. Oxidative markers are common to PBDE exposure, but, in mouse cerebellar granule neurons, the reported potency ranged from pentaBDE-100 > tetraBDE-47 > pentaBDE-99 > hexaBDE-153 >> decaBDE-209 [60,86]. Oxidative damage to voltage-gated sodium channels (VGSCs) has been reported following decaBDE (PBDE 209) exposure in primary cultured rat hippocampal neurons [87]. DNA damage and activated caspase 3 (dependent on ROS production) have been observed in microglia BV2 cells treated with PBDEs [33]. Similarly, Pellacani et al., 2012, reported oxidative DNA damage and cell arrest in a p53-dependent manner in human neuroblastoma cells exposed to tetraBDE-47 and decaBDE-209 (5–20 μmol/L) [88]. The results showed that the effect is more potent for tetraBDE-47 than decaBDE-209 [88]. pentaBDE-99 lead to cytotoxicity in a p53-dependent and calcium- and MAPK-independent form in astroglial cells (human 132-1N1 astrocytoma cells) [89]. In PC12 cells, an undifferentiated neuron cell line’s mitochondrion ultrastructure was severely affected by tetraBDE-47, showing swelling, membrane vacuolation, and neurodevelopmental toxicity. Also, filamentous bumps were observed [90]. pentaBDE-99 altered cytoskeletal proteins, affecting the neurite extension processes [91,92] and neuron growth-associated protein Gap43 [92], both vital processes in brain formation and development. In cultured neural stem cells, tetraBDE-47, pentaBDE-99, decaBDE-209 (0.1–10 µM), and BDE-71 (PentaBDE mixture, 0.5–12.5 μM for 24 h) decreased neurite outgrowth and the differentiation into neurons, as well as proliferation and migration, in a concentration-dependent manner [81,93,94]. BDE-47 caused cytotoxic effects in a concentration-dependent manner in cultured primary rat hippocampal neurons. It has also been shown that tetraBDE-47 disrupts mitochondrial dynamics, neuronal electrical activity, MEK-ERK signaling, and axonal guidance [95,96].

**Figure 1:**
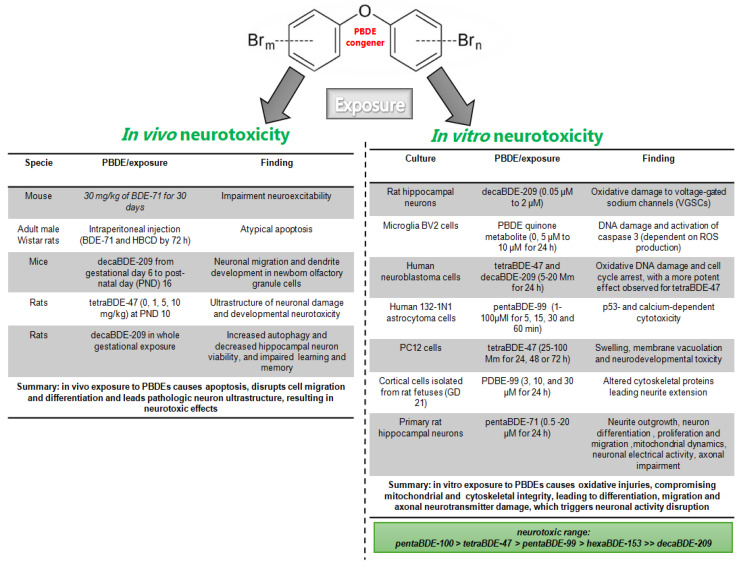
Summary of PBDE in in vivo and in vitro neurotoxic studies. PBDE exposure causes neurotoxicity, characterized by ROS production and oxidative injuries, leading to neuronal integrity and activity disruption observed both in vivo [81, 82, 83, 84, 85] and in vitro models [81, 87, 88, 89, 90, 91, 92, 93, 94, 95, 96].

6-OH-BDE-47, a PBDE metabolite, disrupts calcium homeostasis and neurotransmitter delivery in a more potent form than the parent compound, BDE-47, suggesting that PBDE metabolites are more neurotoxic [97]. Cerebellar cell death by hexabromocyclododecane (HBCD, µM concentrations) was reduced by the NMDA receptor antagonist MK801 (3 µM), the antioxidant alpha-tocopherol (50 µM), and S9 fraction incubation [82]. In summary, these results suggest that structural and ultrastructure alterations contribute to PBDE-induced neurotoxicity during adult, gestational, and early exposure, where PBDE metabolism, oxidative stress, neurotransmitters, and survival signaling are involved (Figure 1).

### 5.3. Pro-Survival Pathway Activated by PBDE Exposure

As an alternative to the damage caused by PBDEs, survival pathways respond to PBDE exposure. Survival signaling is involved in cellular integrity and cognitive processes and is activated by PBDE damage. In this regard, tetraBDE-47, tatraBDE-77, pentaBDE-99, and hexaBDE-153 activate phosphorylated extracellular signal-regulated kinase (pERK1/2), a mitogen-activated protein kinase (MAPK) without significant cytotoxicity in cerebellar granule neuronal cultures. It is known that the MAPK pathway is involved in calcium signaling, critical for neuronal survival, neurodevelopment, and the functioning of the adult nervous system [98]. The MAPK pathway has been suggested as an early mechanism used to avoid the neurotoxic effects of PBDE exposure.

Aldditionally, neurotrophins (NTs) are pro-survival molecules altered by PBDE congeners. In cerebral cortex, primary neuronal cultures of rat cerebellar granule neurons, hexaBDE-153 and pentaBDE-99 treatment significantly decreased the protein contents and mRNA levels of BDNF, GDNF, NGF, NT-3, and NT-4, as well as AchE and ChaT activities [99]. NT signal is crucial to brain development, neuroplasticity, and neurotransmitter delivery, vital to brain functions, and can contribute to PBDE-induced neuron impairments [100,101].

Also, neuropeptides are ubiquitous and dynamic proteins that engage in pro-survival mechanisms, neuroplasticity, behavior, and memory. PBDEs lead to memory impairment with neuropeptides downregulating mRNA transcripts for oxytocin (Oxt) in the bed nucleus of the stria terminalis (BNST) and supraoptic nucleus; vasopressin (Avp) and overexpression receptor Avp1a; and Oxt receptor [102]. Donwregulated neuropeptides can underlie behavior and neurotoxic PBDE effects. Dysregulated neuropeptides may underlie the behavioral and neurotoxic effects of PBDEs, observed in different rodent models, as well as in adults and children (Table 2) [103,104].

In summary, survival signals are activated by the effects of PBDEs. MAPK, neurotrophins, and neuropeptides have a pivotal role in morphology and in the structural, behavioral, and memory impairment caused by PBDEs. This suggests that MAPKs are the initial mechanism underlying the neurotoxic effect of PBDEs, and that the alteration of neurotrophins and neuropeptides is part of the neurotoxic target of PBDEs.

## 6. Brain Epigenetic Effects Associated with PBDEs in Brain Injuries

In humans and other mammals (mainly rodents), normal brain function, as well as glial neurodevelopment, requires stage-specific transcriptional regulation that is linked to cellular signaling. Gene expression is modulated by DNA methylation, histone modification, non-coding RNAs, and microRNAs, essential for glial and neuron differentiation, cell connectivity and migration, synaptic remodeling, neuroplasticity and the process of cognition, memory, and learning [105,106,107]. Epigenetic changes underlie mood and neuropsychiatric diseases [106]. Epigenetic control is the target of ubiquitous and persistent environmental neurotoxics such as polybrominated diphenyl ethers (PBDEs) [108]. Epigenetic alterations associated with PBDEs are observed in reproductive cells (mouse GC-2spd (GC-2) cells, human and rodent sperm samples), human females, breast cancer, and in brain dysfunction in humans and rodents [108,109,110].

Maternal PBDE exposure decreases seven DNA methylation sites in placental tissue and is negatively associated with neonatal anthropometry measures, including the Neurodevelopmental Scale [42,111]. In human cord blood, hypomethylation in TNFα is related to high maternal BDE-47 exposure, suggesting epigenetic reprogramming of the immune response caused by maternal PBDE exposure [112].

Perinatal tetraBDE-47 exposure at 0.2 mg/kg decreased 5-methylcytosine (5mC) in mitochondrial cytochrome c oxidase gene, significantly decreased the methylation of neurodevelopment-related genes (three CpGs in Bdnf and two CpGs in Snca), and significantly increased some DNA methylation stress nuclear genes (five CpGs in Crhr1, two CpGs in Mc2r, and six CpGs in Nr3c1) in the frontal lobe of rats [113]. Global hypomethylation in the brain DNA of female adult mice perinatally exposed to tetraBDE-47 was observed in a genotype-independent manner in a genetically and epigenetically susceptible mouse model with reduced sociability [114]. tetraBDE-47 interfered with aging-related methylated regions in sperm cells in rat perinatal treatments [115]. In the same rat model, tetraBDE-47 led to anticipated changes in small non-coding RNA in younger animals, opposite to what was found in older animals where the variance in the expression of all sncRNAs was decreased [116]. PBDE exposure is harmful to the functioning and neurodevelopment of the embryonic rat cortex [117]. BAF (Brg1-associated factors), a key chromatin remodeling complex, is altered by tetraBDE-47 congener and its hydroxylated metabolite (6OH-BDE-47) [117]. BAF is involved in neurodevelopment and synapse function. In epithelial cells exposed to PBDEs (-47, -99, -209; 10 nM, 100 nM, and 1 μM), the latter induce EZH2 methyltransferase activity, a promoter of inflammation/cancer [118].

The epigenetic changes in DNA methylation and the related molecules described above show the depth of the neurotoxic effect of PBDE exposure. These changes may compromise neurotransmission and lead to neurological effects of PBDEs.

## 7. Neurological Effects of PBDEs

Central and peripheral neurological functions are affected by polybrominated diphenyl ethers (PBDEs), with children being the most vulnerable population (Table 2) [48,53,54,119]. PBDEs are implicated in the prognosis of malignant diseases [120] as well as neurological disorders [121]. Exposure to PBDEs is associated with autism, neurochemical, olfactory, and behavioral alterations [102].

Gestational exposure to tetraBDE-47, pentaBDE-99, or pentaBDE-100 increases the risk of postpartum depression (R = 1.22, 95% CI: 1.03, 1.47) [57]. Neurotoxic levels of PBDEs have been reported in children with environmental exposure to PBDEs [122]. Similarly, exposure to PBDEs (tetraBDE-47 and pentaBDE-99; two main environmental and human sample congeners) has been reported to cause spontaneous, long-term, and lifelong impairments in behavior, memory, and learning [123]. Human prenatal (newborns) and infancy (ages 2, 3, 5, 7, and 9 years) exposure to PBDEs decreases memory ability, with girls being more sensitive to neurotoxic effects [124]. Prenatal or postnatal exposure to decaBDE-209 delays neurological development. PBDE concentrations in milk were significantly and inversely associated with cognitive score (B = −0.007, adjusted R = −0.224, *p* = 0.032). Language was positively correlated with octaBDE-196 (B = 0.096, adjusted R = 0.315, *p* = 0.002) [125].

In vivo models have shown that early, acute, single exposure to tetraBDE-47 (1, 10, or 30 mg/kg) on PND 10 leads to hyperactivity at both 2 and 4 months in mice [126]. In adult rats, decaBDE-209 was reported to decrease spatial ability and the activation/expression of two hippocampal proteins related to plasticity, NR1 and NR2B [127]. Neonatal exposure to decaBDE-209 (1, 10, or 20 mg/kg body weight) once daily from PND 5 to 10 resulted in decreased spatial localization and working and reference memory (Morris water maze, working and eight-arm radial maze). Concurrently, synaptobrevin 2, syntaxin 1A, SNAP-25, and synaptophysin mRNA were decreased in the hippocampus, showing impairment of the synaptic ultrastructure [128]. Additionally, in vivo hippocampal LTP, a type of cellular memory, was reduced after exposure to 6.8 mg (14 micromol)/kg body weight (bw) tetraBDE-47 in adult C57Bl/6 mice, treated with a single oral dose of tetraBDE-47 on PND 10 (growth spurt period) [129]. In vitro and in vivo exposure to pentaBDE-99 increased cGMP delivery, second messaging, and the strength of LTP in the extracellular space [130]. Indeed, mice showed a postsynaptic downregulation of NMDA subunits NR2B and GluR1 and activation of Ca^(2+)^/calmodulin-dependent protein kinase II, critical in the LTP/memory process (alphaCaMKII) [129].

In sumary, adult human exposure to PBDEs decreases memory capacity and can lead to neuropathologic conditions such as depression. Similarly, in rodent models, exposure to PBDEs reproduced the neuroxic effects observed in humans, such as cognitive impairment (spatial memory and motor impairments).

PBDEs negatively affect human memory. Gestational and early exposure to PBDEs is inversely correlated with intelligence and memory scores. In rodent models, it was observed that exposure to PBDE congeners led to a decrease in neurotransmitters and affected neuroplasticity, memory pathways, and spatial memory. This suggests that the neurotransmitter systems involved in behavior, neurodevelopment, and memory are putative neurotoxic mechanism in PBDEs.

## 8. Neurotransmitters Altered by Exposure to PBDEs

### 8.1. Glutamatergic Impairment by Exposure to BPDEs

Glutamatergic neurotransmission is central to many vital phenomena, such as neurodegeneration, neuroprotection, and toxicity to different substances present in the environment, such as polybrominated diphenyl ethers (PBDEs). The glutamate system can show PBDE-mediated neurocognitive deficits that arise from the frontal cortex [81]. In the same vein, it is proposed that these alterations occur through the regulation of neurosteroids (pregnenolone, dehydroepiandrosterone, progesterone, and allopregnanolone disruption) [131]. Thyroid hormones have a role in mood disorders, cognitive loss, and psychiatric symptoms [132].

Microdialysis analysis showed increased glutamate–nitric oxide-cGMP in the brains of freely moving rats prenatally exposed to BDE-99; these results were reproduced in a primary culture of PBDE-exposed rats and PBDE-exposed control neurons [130]. Glutamatergic subunit receptor subunit NR1 expression was increased in mice perinatally intoxicated with decaBDE-209 (20 mg/kg at PND 3–10) at the time that memory impairment was observed [133]. In adult rats, decaBDE-209 decreased spatial abilities and altered hippocampal glutamatergic proteins, subunits NR1, NR2B, and GluR1, and the phosphorylation of the NR2B subunit at Ser1301 (p-NR2B Ser1303) and the GluR1 subunit at Ser831 (p-GluR1 Ser831) was decreased [127]. Immunohistochemistry showed that tetraBDE-47, 0.1, 0.5, and 1 mg/kg every 30 days, significantly decreased mRNA expressions of NR(1), NR(2)B, and Glu in the CA1, CA3, and dentate gyrus areas of the hippocampus in adult male Sprague Dawley rats with memory deficits [134].

tetraBDE-47 cytotoxicity in mice cerebellar granule neurons was dependent on oxidative stress and calcium levels related to the activity of ionotropic glutamate receptors (NMDA and AMPA/Kainate receptors); tetraBDE-47 led to increased extracellular glutamate levels, but the pharmacological inhibition of NMDA and AMPA/Kainate receptors prevented the calcium overload, oxidative stress, and neurons death [80].

The described results involve neuron death and damage following exposure to PBDEs, mediated by glutamatergic neurotransmission through decreases in the expression and activity of the NMDA receptor.

### 8.2. GABAergic Impairment by BPDE Exposure

The inhibitory neurotransmitter γ-aminobutyric acid (GABA) counteracts the action of brain excitability, most of which is glutamatergic. GABA is also affected by tetraBDE-47 and its metabolite 6-OH-PBDE-47 in a mixture or alone [135], as well as by decaBDE-209 [131]. In addition, in frontal cortex cultures, PBDEs influenced GABAergic neurotransmission; GAD67, vGAT, vGlut, and the GABA(A) 2α receptor subunit were altered, and the cortex appeared to be the specific target region of the PBDE neurotoxicity [81].

The specificity of the α1 subunit of gamma-aminobutyric acid receptor A (GABAAR) mRNA was decreased, but that of the β2 and γ2 subunits of GABAAR was increased in the hippocampus following the exposure of offspring to PBDEs during pregnancy and lactation, affecting learning and memory formation [131]. A decrease in GABA levels was observed in Caenorhabditis elegans exposed to Tetrabromobisphenol-A-bis(2,3-dibromopropyl ether) at relevant environmental and biological concentrations (0 to 100 μg/L) [136]. A decrease in GABA expression is another molecular, neurobehavioral contributor and endpoint of neurotransmission impairment following PBDE exposure.

### 8.3. Acetylcholine Impairment by Exposure to PBDEs

PBDEs have been hypothesized to affect locomotor function via the cholinergic system. Studies in mice exposed to decaBDE-209 (0, 1.4, 6.0, and 14.0 μmol/kg b.w.) on PND 3 showed habituation to novelty, impaired learning and memory, and increased tau neuroprotein and susceptibility of the cholinergic system (spontaneous behavior) in the adult mice treated (2–7 months) [137]. Oral neonatal exposure to pentaBDE-99 [138], octaBDE-203, and hexaBDE-153 resulted in impaired learning and memory, as well as spontaneous behavior, in both mice and rats. Mice showed reduced numbers of nicotinic receptors in the hippocampus, and these effects worsened with age in 2-, 4-, and 6-month-old mice [139,140]. pentaBDE-99 and Tetrabromobisphenol A (TBBPA) (brominated flame-retardant type) decreased nicotinic cholinergic receptor ligand binding sites in the frontal cortex. Radioactivity assays show that the TBBPA of TBBA peaked earlier and decreased faster than pentaBDE-99. Indeed, calcium/calmodulin-dependent protein kinase II (CaMKII), growth associated protein-43 (GAP-43), and synaptophysin were affected by neonatal exposure to pentaBDE-99 (21 μmol/kg body weight), only in pentaBDE-99 exposure [141]. Changes in cholinergic protein expression can be related to the behavioral disturbances observed in studies of different PBDE congeners.

### 8.4. Dopaminergic Impairment by PBDE Exposure

Dopamine, a catecholamine that is altered in Parkinson’s disease (PD), is another pathway implicated in PBDE-induced neurotoxicity. In rodents, exposure to PBDEs led to a decrease in the dopaminergic system in vitro and in vivo. In this vein, exposure of the catecholaminergic cell line SK-N-SH to 0–10 μM PBDEs decreased cell growth and viability. Male mice exposed to hexabromocyclododecane (HBCDD) showed reduced levels of presynaptic dopaminergic proteins, including TH, COMT, MAO-B, DAT, VMAT2, and alpha-synuclein [142]. In vivo exposure to DE-71 led to significant reductions in dopamine, dopamine transporter (DAT), and VMAT2 in striatum [143].

In vivo exposure to 25 mg/kg of HBCDD for 30 days showed significant reductions in the striatal transporter and in storage, damaging the dopamine circuit [144]. In PC12 cells, HBCD (0–20 microM) inhibited depolarization-evoked [Ca^(2+)^](i) and dose-dependent neurotransmitter release [145]. These results demonstrate the significant risk of alteration that environmental exposure to HBCDD poses in PD and other neuropathologies.

### 8.5. Nitric Oxide Altered by Exposure to PBDEs

Nitric oxide (NO) is a retrograde gaseous neurotransmitter involved in synaptic strength, olfactory recognition, learning, and memory pathologies [146,147,148,149]. Additionally, in human dementias like Alzheimer disease, an increase in NO prevents cognitive impairment [149]. NO is a target of PBDE compounds [150,151]. NO is critical for the formation of synaptic connections [152]. NO is a promiscuous molecule and highly reactive radical that interacts with ion channels and receptors (NMDA) through S-nitrosylation. At low concentrations, NO mediates the regulation of transcription factors involved in normal neurotransmission or vasodilatation, but at higher concentrations it mediates neurotoxic actions and activate kinases such as PKA [153]. NO is enzymatically produced in the hippocampus and cortical neurons in a calcium-N-metil-D-Aspartate dependent-manner, with neurotransmission being altered by PBDEs [127,130,133,134].

NO synthesis is activated via an excitatory pathway; NO diffuses from neuron to neuron and then acts directly on soluble guanylyl cyclase (GC) to form the second messenger, cyclic guanosine monophosphate (cGMP). cGMP-dependent protein kinase (PKG) signaling has a key role in retrieval memory [149,154].

In fact, in the memory process, the NO system participates in cardiovascular and osmotic homeostasis [149]. In this sense, vasopressinergic effects are essential for cardiovascular function and long-term cGMP potentiation (LTP, a model mechanism of cellular learning and memory) and long-term depression (LTD, cellular memory loss). Vasopressin is negatively regulated by NO, participates in cognition, and influences complex social behaviors [155,156].

The epidemiological observations seen following exposure to PBDEs are associated with an increase in arterial pressure and a decrease in attention, intelligence, and neurodevelopment measures in children and young adolescents [56,157,158].

In animal experiments, it was reported in a hyperosmotic treatment that gestational PBDEs caused significant changes in systolic blood pressure in a plasma vasopressin-independent manner [159]. The Leon-Olea group reported that perinatal exposure to PBDEs (penta commertial mixture BDE-71, 30 mg/kg/day at gestational day 6 to PND 21) impaired the vasopressin system in a sex-independent manner [151]. In the same vein, perinatal exposure (from GD 6 to PND 21) to pentaBDEs mixture (BDE-79, 0, 1.7, or 10.2 mg/kg/day) disturbed NO, a vasopressinegic regulator, leading to long-term osmoregulation deficit in adult rat males [121]. An endothelial cell culture exposed to PBDEs showed oxidative damage and reticular stress, autophagy, and apoptosis, suggesting that PBDEs show a risk of cardiovascular diseases [160]. In summary, exposure to PBDEs compromises molecules such as NO, a regulator of central and peripheral processes, leading to neurological effects accompanied by systemic illness.

## 9. Conclusions Remarks and Perspectives

**Figure 2:**
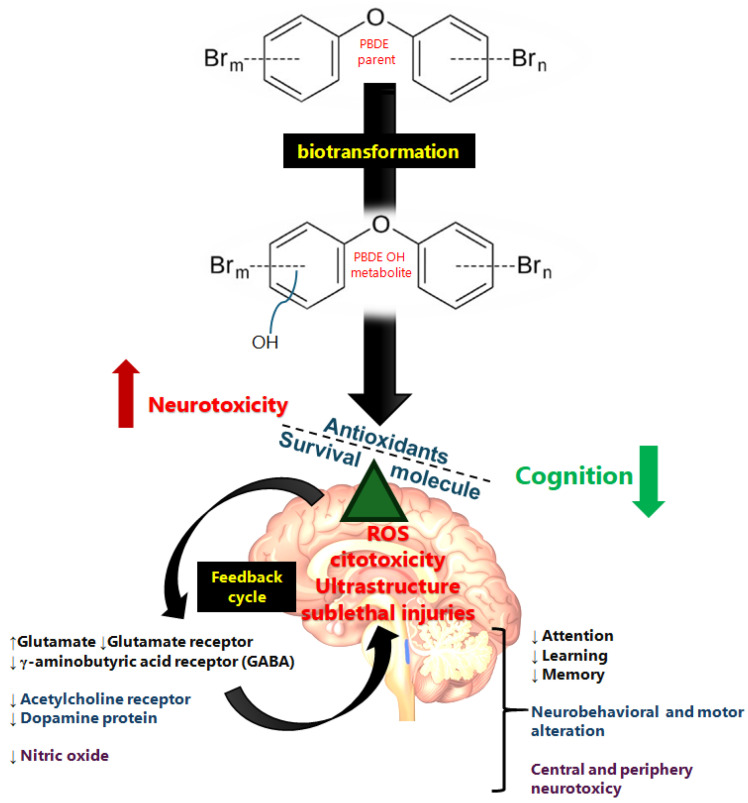
Summary schematic of PBDE neurotoxic mechanism. The lipophilic compound crosses the membrane and arrives in the brain, where it has a direct effect though ROS derivatives of the biotransformation of PBDEs, leading to ultrastructure and biochemical neurotransmission alterations. Glutamate, γ-aminobutyric acid, dopamine, acetylcholine, and nitric oxide are involved in the neurotoxic effects of PBDEs (apoptosis, synaptic ultrastructure, and neurotransmitter alteration) observed in vulnerable populations such as children. The direction of the arrows indicates the increase (↑) or decrease (↓) in each case.

Polybrominated diphenyl ethers (PBDEs) are synthetic neurotoxic molecules of epidemiological interest. These compounds are persistent, lipophilic, and stable in the environment and are mobilized, occurring in water, soil, air, and food, the main sources of exposure to PBDEs. PBDEs are a complex family used in industry in the form of mixtures and they are stored in mammalian tissues. Follow-up studies in humans have reported them in serum, milk, and biota. Different studies suggest that they undergo biotransformation and that the metabolites are more toxic than the parent compounds. Developing organisms are more susceptible to the brain-toxic effects of PBDEs and their outcomes are more severe; sex-based susceptibility is inconclusive. In summary, these results suggest that structural and ultrastructural alterations contribute to PBDE-induced neurotoxicity during adult, gestational, and early life exposure, involving PBDE metabolism, oxidative stress, and neurotransmitters. Survival pathways are activated early upon exposure to PBDEs. PBDEs exert a toxic mechanism related to oxidative stress that compromises synaptic ultrastructure, brain morphology, and neuronal viability, in addition to processes such as migration, differentiation, neuroplasticity, neurodevelopment, and neurotransmission. PBDE-induced neurotoxicity is accompanied by alterations in the neurotransmitter system, increased oxidative molecules, transport, and neurotransmitter receptors, and impaired intracellular signaling. The glutamatergic and GABAergic systems are the main neurotransmitters decreased by PBDEs, which may underlie the neurological effects. Furthermore, disruptions to dopamine and acetylcholine are suggested to lead to neurobehavioral/motor impairment, and nitric oxide is involved in the central and peripheral PBDE neurotoxicity observed primarily in environmental exposure to PBDE congeners in children. Oxidative stress is suggested to contribute to subtle structural alterations in synapses and neurotoxicity in the developing and adult brain of PBDE-exposed animals. However, the overactivation of neurotransmitters increases the neuronal ROS that are part of the toxic feedback loop of PBDEs, leading to the neurological effects observed in humans exposed to PBDEs (Figure 2).

Moreover, these compounds induce a deep change in cellular phenotypes because they alter the epigenetic process and underlie the long-term and irreversible neurotoxic effects. To date, there is no safe PBDE level accepted worldwide. The continuous monitoring of human and biota exposure to PBDEs is necessary to prevent public health risks and limit the contact of multiple organs to neurotoxic mixtures and their PBDE derivatives.

## Figures and Tables

**Table 1:** Different properties of main polybrominated diphenyl ethers in biological samples.

Family	Water Solubility	Clearance	Mobilization	Stored	Toxicity
tetraBDE pentaBDE hexaBDE heptaBDE octaBDE decaBDE	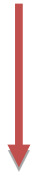	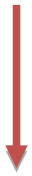	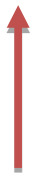	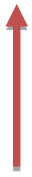	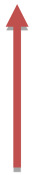

Example of BDEs congener type: tetra-(BDE-66), penta-(BDE-47, -99, -100, and -153), octa-(mixtures of hexa to decaBDE), and deca-(BDE-209). The direction of the arrows indicates the increase (↑) or decrease (↓) in each PBDE group; tetraBDE to hexaBDE show more mobilization, storage, and toxicity.

## Data Availability

Not applicable.

## Abbreviations

The following abbreviations are used in this manuscript:

PBDEs	Polybrominated diphenyl ethers
BDE	Brominated diphenyl ethers
Br	Bromine
BDE-17	2,2′,4-Tribromodiphenyl ether
BDE-28	2,4,4′-Tribromodiphenyl ether
BDE-47	2,2′,4,4′-Tetrabromodiphenyl ether
BDE-66	2,3′,4,4′-Tetrabromodiphenyl ether
BDE-77	3,3′,4,4′-Tetrabromodiphenyl ether
BDE-85	2,2′,3,4,4′-Pentabromodiphenyl ether
BDE-99	2,2′4,4′,5-Pentabromodiphenyl ether
BDE-100	2,2′,4,4′,6-Pentabromodiphenyl ether
BDE-138	2,2′,3,4,4′,5′-Hexabromodiphenyl ether
BDE-153	2,2′,4,4′,5,5′-Hexabromodiphenyl ether
HBCD	1,2,5,6,9,10-Hexabromocyclododecane
BDE-154	2,2′,4,4′,5,6′-Hexabromodiphenyl ether
BDE-183	2,2′,3,4,4′,5′,6-Heptabromodiphenyl ether
BDE-209	2,2′,3,3′,4,4′,5,5′,6,6′-Decabromodiphenyl ether
